# Impact of COVID-19 on the pre and post analytical clinical laboratory testing processes- A performance evaluation study using six sigma

**DOI:** 10.1016/j.amsu.2021.102842

**Published:** 2021-09-09

**Authors:** Sibtain Ahmed, Farhat Jahan, Muhammad Umer Naeem Effendi, Farooq Ghani

**Affiliations:** a Department of Pathology and Laboratory Medicine, Aga Khan University, Stadium Road, P.O. Box 3500, Karachi, 74800, Pakistan; b Section of Clinical Chemistry, Department of Pathology and Laboratory Medicine, Aga Khan University, Stadium Road, P.O. Box 3500, Karachi, 74800, Pakistan; c Pathology and Laboratory Medicine, Aga Khan University, Stadium Road, P.O. Box 3500. Karachi, 74800, Pakistan

**Keywords:** Clinical laboratory, COVID-19, Impact, Quality indicators, Pakistan

## Abstract

**Background:**

The aim of this study was to determine the impact of COVID-19 pandemic on the total testing process using six sigma metrics based on a comparison of pre and during pandemic periods.

**Material & methods:**

The study duration was over 12 months, 6 months before and 6 months after the COVID-19 onset in Pakistan in March 2020 after the recognition of the first case, using quality indicators (QIs). QIs were chosen from a model of QIs recommended by the CAP. Analysis was performed using Six Sigma calculators and QIs were expressed in percentage (%) and Sigma value were computed. Three levels of performance quality i.e. 25th, 50th, and 75th percentile were derived, being best, common and worst performance respectively. Between-group differences were tested using the Mann-Whitney's *U* test.

**Results:**

The median defect percentages of these QIs ranged from 0% to 0.27% for the pre-pandemic period and 0% to 0.13% for the during pandemic period. Meanwhile, sigma values of the majority of the QIs were all above 4.0σ during the pre and the pandemic times. For the pre-analytical phase, sigma scores declined for 1 QI, improved for 3 QIs and remained same for 2. In the post analytical phase, no change in sigma metrics was noted for critical values notification. Considerable increase in defect percentage of inappropriate turnaround times was noted.

**Conclusion:**

The emergency preparedness proved to be fruitful as depicted by exceptional performance on the sigma metrics for most Qis both prior to and during the pandemic. The pre-analytical and the post analytical phases, being the most error sensitive requires strict vigilance.

## Highlights

• The growing diagnostic demands in the COVID-19 era have impacted clinical laboratory testing processes.
• Quality indicators (QIs) are considered as the fundamental tools of maintaining quality in laboratory systems.
• Modern quality-management tools such as Six Sigma was utilized for performance evaluation.
• Excellent performance on the sigma metrics for most QIs both prior to and during the pandemic were recorded.

## 1 Introduction

The Corona virus disease 2019 (COVID-19) pandemic affected almost every nation across the globe, impacting more than 20 million lives alongside services and economic productivity [1]. The pandemic triggered an unexpected global emergency which has had huge impact on many organizations including health structure, health care professionals and clinical laboratories [2]. The part played by laboratory medicine during a pandemic has been widely recognized [3]. A comprehensive summary of previous researches strengthens the fact that the diagnosis of COVID-19 would not have been possible without the laboratory services, either by detecting the pathogen through reverse transcriptase-polymerase chain reaction (RT-PCR), or quantifying antibody response via immunological based techniques [4]. Hence, laboratories have a crucial part to play during an infectious outspread from the time of diagnosis till surveillance.

While the pandemic has highlighted the significance of laboratory medicine in healthcare setups, laboratory testing is altogether an intricate process [5]. Each of the various steps involved total testing process can result in errors and they can have a huge impact on patient prognosis [6]. In laboratory practice, total testing process is divided into three crucial steps: pre-analytical, analytical and post-analytical [7]. Quality indicators are considered as the fundamental tools of maintaining quality in laboratory systems that can be gauged to assess each step of total testing process. The utilization of quality indicators in clinical laboratory allows determining error incidence and diminishing or arresting patient safety errors [8].

This COVID-19 pandemic has clearly exhausted the laboratories resources with obstacles such as shortage of staff, conveyance issues, short supply of personal protective equipment (PPE), delayed shipments of necessities, and particularly, fear and anxiety amongst healthcare workers. The preparations to tackle such crises are never mentioned in the books of laboratories especially for resource constrained setups in developing world.

During the ongoing pandemic, the healthcare professionals were made to follow additional caution and wear recommended PPE. Accreditation and regulatory bodies are increasingly emphasizing laboratories to go beyond analytical quality and focus on the pre- and post-analytical phases, where most errors arise that can influence clinical care. Furthermore, the specimen collection and transport logistics were also considerably unalike from the pre-pandemic times [7,8]. The impact of these changed protocols for specimen collection, packaging and transport on the total testing process is not widely established. The purpose of this study was to determine the impact of COVID-19 pandemic on the pre and post analytical testing process using six sigma metrics based on a comparison of pre and pandemic periods.

## 2 Material & Methods

This retrospective observational study was conducted at the Section of Clinical Chemistry, Department of Pathology and Laboratory Medicine at Aga Khan University Hospital (AKUH), Karachi, Pakistan. The clinical laboratory is the largest reference laboratory catering a country wide population of 240 million with its growing network of more than 280 phlebotomy stations and stat laboratories spread across Pakistan. The laboratory operates to highest standards of quality and was the first to be accredited by Joint Commission International Accreditation (JCIA) and the only lab with College of American Pathologist (CAP) accreditation in Pakistan.

This study was done to evaluate the repercussions of the pandemic on the quality of the test process. The study duration was 12 months, 6 months before and 6 months following the COVID-19 onset in Pakistan in March 2020 after the recognition of the first case, using Q6 Is. Data was retrospectively retrieved from the electronic laboratory information management system. At our center, the preanalytical, analytical, and postanalytical processes are regularly monitored and recorded using QIs and presented in monthly departmental quality management committee meeting for appropriate actions if necessary. Quality indicators were chosen from a model of QIs recommended by the CAP [9]. QIs were categorized in accordance with the main TTP phases as shown in Table 1.

**Table 1:** QIs representing pre and post analytical phase from pre- and during pandemic periods.

	Quality Indicators	Period	Defect percentage (%)			Sigma values			*p-value*
			25th	50th	75th	25th	50th	75th	
**Pre-Analytical** **Phase**	**Percentage of number of hemolyzed samples/total number of samples**	**Pre-pandemic**	0.05	0.05	0.06	4.8	4.7	4.7	0.16
		**During pandemic**	0.05	0.06	0.06	4.7	4.7	4.7	
								
	**Percentage of number of QNS samples/total number of samples**	**Pre-pandemic**	0.01	0.02	0.02	5	5	4.9	1.00
		**During pandemic**	0.01	0.01	0.02	5	4.9	5.1	
								
	**Percentage of number of empty container/total number of samples**	**Pre-pandemic**	0	0	0	6	6	6	0.93
		**During pandemic**	0	0	0	6	6	6	
								
	**Percentage of number of bad barcode samples/total number of samples**	**Pre-pandemic**	0	0	0.01	5	4.9	4.4	0.81
		**During pandemic**	0.005	0.03	0.04	4.6	4.7	4.5	
								
	**Percentage of number of without barcode sample/total number of samples**	**Pre-pandemic**	0.16	0.23	0.27	4.5	4.4	4.3	0.87
		**During pandemic**	0.07	0.1	0.13	4.7	4.6	4.4	
								
	**Percentage of number of leaking container/total number of samples**	**Pre-pandemic**	0	0	0	6	6	6	0.93
		**During pandemic**	0	0	0	6	6	6	
**Post-Analytical** **Phase**	**Percentage of number of critical results informed within time/total number of samples**	**Pre-pandemic**	0	0	0	6	6	6	0.93
		**During pandemic**	0	0	0	6	6	6	
								
	**Percentage of number of STAT samples/total number of samples**	**Pre-pandemic**	0.003	0.003	0.004	4.4	4.5	4.3	0.37
		**During pandemic**	0.006	0.008	0.009	4.4	4.4	4.6	

Analysis was performed using Six Sigma calculators and QIs were expressed in percentage (%) and sigma value were computed [10]. Three levels of performance quality i.e., 25th, 50th, and 75th percentile was derived, being best, common and worst performance respectively as described previously by Sciacovelli L et al. [11]. Between-group differences were tested using the Mann-Whitney's *U* test taking a p-value of <0.05 as statistically significant difference. The Statistical Package for Social Sciences (SPSS) software program (v.22; IBM, Armonk, NY) and a P < 0.05 was considered as statistically significant for all analyses. This work has been reported in line with the STROCSS criteria [12].

## 3 Results

A total of 2,528,589 specimens were screened of which 999,437 (40%) were received during the pandemic phase. A total of 30,926 critical results were informed out of which 12,118 (39%) were during the pandemic phase. Moreover, 214,884 stat results were processed of which 83,065 (39%) were during the pandemic period. The error rates for all QIs were regularly monitored, against established benchmarks, adopted from the Q-Probes and Q-Tracks studies from CAP. A review of the records revealed that all the Qis were well within the respective benchmarks [13].

Defect percentages and sigma values related to pre-analytical, analytical and post-analytical phase are depicted in Table 1. The median defect percentages of these QIs ranged from 0% (leaking containers) to 0.27% (manual labeling, without barcode) for the pre-pandemic period and 0% (leaking containers) to 0.13% (without barcode samples) for the during pandemic period. The QIs were monitored through rigorous and vigilant practices at the receiving and processing bench housed by a team of technologists led by a charge technologist. As correct patient identification and correct specimen labeling are recognized cardinal safety goals by CAP, without barcode samples with manual entries were visually scanned and segregated. The entries were re-verified from the test requisition slips and barcodes generated and applied for optimal further processing and analysis.

For the 1 QI (bad barcode samples) defect percentages increased with a decrement in sigma values during the pandemic compared to the pre-pandemic phase. On the contrary, sigma metrics improved, and defect percentages went down for 2 QIs (number of insufficient quantity (QNS) samples, number of without barcode samples). Whereas sigma values for 3 QIs (hemolyzed samples, leaking containers, empty containers) were almost the same before and during the pandemic.

**Fig. 1:**
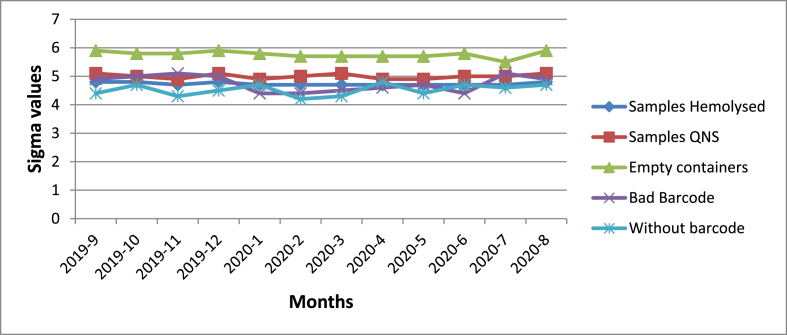
Sigma trend of quality indicators.

Amongst the 2 QIs related to the post analytical phase, no change in sigma metrics was noted for critical values notification. While there was a considerable increase in defect percentage of inappropriate turnaround times with lower sigma scores compared to the pre pandemic era as depicted in Table 1. However, as the difference between the pandemic and pre pandemic era was not significant, the p-values calculated were not statistically significant. Meanwhile, sigma values of the majority of the QIs were all above 4.0σ before and after the onset of the pandemic as depicted by the graphical trends in Fig. 1.

## 4 Discussion

This study is the first to assess the impact of the pandemic on the testing processes at a high-volume clinical chemistry laboratory in Pakistan, a lower middle-income country hardest hit by the pandemic. The sudden rise in cases, increasing demands of prognostic biomarkers including ferritin, lactate dehydrogenase, procalcitonin, interleukin-6 and c-reactive protein and different coping strategies adopted, led to enhanced workload and pressure on laboratory staff, making them prone to errors [14,15]. As anticipated, a mixed trend was noted for the QIs on comparison between the pre and during pandemic phases, with improvements in some areas to regression in others.

From a laboratorian's perspective, the pre-analytical errors account for up to 70% of all mistakes made in laboratory diagnostics [16]. It is this phase that can highly influence the test results in turn impacting medical decisions. However, in this study only one QI belonging to the pre-analytical phase showed a decline whereas the sigma scores for the others QIs either remained unchanged or showed an improving trend. Our results are in contrast to Tapasyapreeti M et al., who have reported that preanalytical errors and resultant blood specimen rejection rate at a clinical laboratory in India have significantly increased due to changed logistics [17]. This comparison substantiates the impact of contingency measures taken by our laboratory to facilitate logistics and staff shortages managed appropriately even during the strict lock down period, that prevented any major decline in the sigma scores in this vulnerable pandemic phase.

In the post analytical phase, the critical result reporting maintained the sigma benchmark of the pre-pandemic era. However, few stat delays were encountered during the pandemic phase. Our findings are in contrast with Eren F et al. and colleagues who have reported high percentage of critical reporting delays during the pandemic at a clinical laboratory in Ankara [18]. The few instances of stat delays were linked with delayed specimen transport from clinical care areas, which may be related to staff shortages.

There were certain limitations of our study, notably more than 15 QIs could be used to evaluate the testing processes, however the most significant ones that can impact lab performances were examined in this study. Moreover, the performance of external quality assessments and proficiency testing was not assessed on the sigma metrics, because of the difference in time intervals of different surveys which may influence sigma calculations.

## 5 Conclusion

Identification of lab errors, rectification and vigilance serve as pillars of total quality management. The emergency preparedness and strategies adopted amidst the crisis situation proved to be fruitful as depicted by exceptional performance on the sigma metrics for most QIs in the high-volume reference laboratory in Pakistan. The pre-analytical and the post-analytical phases, being the most error sensitive requires strict vigilance to maintain good quality assurance in a clinical laboratory.

## Ethical approval

Not applicable as the research work is based on review of quality indicator data of laboratory and does not involve intervention/interaction with human subjects or related information.

## Funding

None.

## Author contribution

SA performed the literature search, data analysis and write-up of the work in the first draft. FJ was involved in data retrieval and analysis. MUNE assisted in manuscript writing. FG conceived the idea, coordinated the writing of the paper and reviewed the final draft. All the authors have accepted responsibility for the entire content of this submitted manuscript and approved submission.

## Registration of research studies

Not applicable.

## Guarantor

Dr Farooq Ghani.

Associate Professor, Department of Pathology and Laboratory Medicine.

Aga Khan University.

Stadium Road, Karachi, Pakistan. P.O. Box 74800.

Telephone: 021-34861927.

Email: farooq.ghani@aku.edu.

## Consent

Not applicable.

## Provenance and peer review

Not commissioned, externally peer-reviewed.

## Declaration of competing interest

None.
